# Comparative Proteomics Analysis for Elucidating the Interaction Between Host Cells and *Toxoplasma gondii*

**DOI:** 10.3389/fcimb.2021.643001

**Published:** 2021-05-13

**Authors:** Hui Sun, Jin Li, Longjiang Wang, Kun Yin, Chao Xu, Gongzhen Liu, Ting Xiao, Bingcheng Huang, Qingkuan Wei, Maoqing Gong, Jianping Cao

**Affiliations:** ^1^ Shandong Institute of Parasitic Diseases, Shandong First Medical University & Shandong Academy of Medical Sciences, Jining City, China; ^2^ National Institute of Parasitic Diseases, Chinese Center for Disease Control and Prevention (Chinese Center for Tropical Diseases Research), National Health Commission of People's Republic of China Key Laboratory of Parasite and Vector Biology, World Health Organization Collaborating Centre for Tropical Diseases, Shanghai, China; ^3^ The School of Global Health, Chinese Center for Tropical Diseases Research, Shanghai Jiao Tong University School of Medicine, Shanghai, China

**Keywords:** *Toxoplasma gondii*, host–parasite interaction, TMT, comparative proteomics, differentially expressed proteins

## Abstract

*Toxoplasma gondii*, a representative model organism belonging to the phylum Apicomplexa, can infect almost all warm-blooded organisms, including humans. The invasion of host cells *via* host–parasite interaction is the key step for *T. gondii* to complete its life cycle. Herein we performed tandem mass tag analysis to investigate global proteomic changes in host cells (human foreskin fibroblasts, HFFs) [HFFs infected with *T. gondii* (HT) *vs*. HFFs (H)] and *T. gondii* [HT *vs*. *T. gondii* (T)] during intracellular infection. Overall, 3477 and 1434 proteins were quantified, of which 375 and 1099 proteins were differentially expressed (adjusted p-value < 0.05 and >1.5 or <0.67-fold change) in host cells and *T. gondii*, respectively. *T. gondii* invasion relies on the secretion of numerous secretory proteins, which originate from three secretory organelles: micronemes, rhoptries, and dense granules. In the HT *vs*. T group, few secretory proteins were upregulated, such as microneme proteins (MICs: MIC6, MIC10), rhoptry bulb proteins (ROPs: ROP5, ROP17), and dense granule proteins (GRAs: GRA4, GRA5, GRA12). In contrast, dozens of known secretory proteins were significantly downregulated in *T. gondii*-infected HFFs. In HFFs, gene ontology and Kyoto Encyclopedia of Genes and Genomes pathway analyses revealed a large number of differentially expressed proteins (DEPs) enriched in metabolic processes and immune-associated signaling pathways, such as NF-κB, cAMP, and Rap1 signaling pathways. Further, in case of *T. gondii*, DEPs were involved in ribosome biogenesis, citrate cycle, and galactose metabolism, indicating that cell biosynthesis and metabolism of *T. gondii* were altered after host cell invasion. These findings reveal novel modifications in the proteome of host cells as well as *T. gondii*, helping us better understand the mechanisms underlying host–parasite interaction.

## Introduction

Toxoplasmosis is a life-threatening opportunistic infection caused by the protozoan parasite *Toxoplasma gondii*, an obligate intracellular organism. Around one-third of the global population is considered to have a latent infection of *T. gondii* (Montoya and Liesenfeld, 2004). *T. gondii* is a representative model organism of the phylum Apicomplexa that contains many important parasites of humans and animals, including *Plasmodium* spp., *Eimeria* spp., *Neospora*, and *Cryptosporidium* (Zhang et al., 2012; Janouškovec et al., 2015). *T. gondii* can infect almost all warm-blooded organisms, including humans, and can exist in various tissues and organs of infected hosts. Moreover, toxoplasmosis can be fatal in immunocompromised patients, such as those with AIDS and those undergoing immunosuppressive chemotherapy (Kim and Weiss, 2008; Flegr, 2013; Torrey and Yolken, 2013). In addition, *T. gondii* can often cause adverse pregnancy outcomes, such as abortion and preterm labor, and even if pregnant women can successfully deliver, surviving offspring often show serious neurological and ocular sequelae (Robert-Gangneux et al., 2011; Jiao et al., 2017).

*T. gondii* actively invades host cells and can infect and replicate within almost any nucleated cell, which suggests that the pertinent mechanism is precise as well as effective. Host cell invasion is pivotal for *T. gondii* to complete its life cycle and is a consequence of host–parasite interaction (Hakimi et al., 2017). *T. gondii* participates in the signal transduction of host cells, making them prone to invasion. The invasion of host cells by *T. gondii* can be achieved in merely 15–20 s and relies on the actin–myosin motor complex and multiprotein complexes secreted by specialized organelles at its apical end, such as micronemes, rhoptries, and dense granules (Rabenau et al., 2001; Frénal et al., 2010; Swierzy et al., 2017). These secretory organelles have a crucial function in host cell invasion and parasitophorous vacuole establishment (Soldati et al., 2001). The microneme protein (MIC) plays an important role in the recognition, adhesion, and attachment of *T. gondii* to host cells during the early stage of invasion. Rhoptry proteins, comprising rhoptry neck proteins (RONs) and rhoptry bulb proteins (ROPs), are secreted during invasion and are believed to contribute to parasitophorous vacuole formation. Further, dense granule proteins (GRAs) participate in the modification of parasitophorous vacuole and its membrane, facilitating survival and replication of the organism.

The host–parasite response to an infection and the identification of host proteins that interact with *T. gondii* proteins should help in elucidating the mechanism of infection and virulence at the tachyzoite stage. Previous studies have majorly used two-dimensional electrophoresisto detect proteomic changes in human foreskin fibroblasts (HFFs) or *T. gondii* (Cohen et al., 2002; Nelson et al., 2008); however, this method has a relatively low throughput for protein identification or quantification. Herein we performed tandem mass tag (TMT) analysis to investigate global proteomic changes in both host cells and *T. gondii* during intracellular infection. We report novel insights into the proteomics landscape of *T. gondii* before and after infection of host cells and also report data pertaining to modifications in the host cell proteome during *T. gondii* infection, with the aim of elucidating the complex relationship between host cells and parasites.

## Material and Methods

### Parasite, Cell Culture, and Cell Invasion Experiments

*T. gondii* RH strain tachyzoites were maintained in HFFs at 37°C and 5% CO_2_ in Dulbecco’s modified Eagle’s medium (catalog no. SH30022.01B, Hyclone, China) supplemented with 10% fetal bovine serum (catalog no. 10099133C, Gibco, USA), 100 IU/mL penicillin, and 100 μg/mL streptomycin (catalog no. P1400, Solarbio, China). Tachyzoites were harvested from monolayer HFFs and purified using a 5-μm filter. They were then washed using phosphate-buffered saline and counted with trypan blue stain.

For cell invasion experiments, tachyzoites were added to HFF monolayers (approx. 85% confluency) at an infection ratio of 3:1. Tachyzoites that had not invaded the cells were washed away with phosphate-buffered saline after 2 h. The cells were harvested at 24 h when approximately 80% of them appeared infected. The control groups included an equal number of non-infected cells and an equal number of *T. gondii* tachyzoites. All cells were washed using cold phosphate-buffered saline, centrifuged at 400 g for 10 min, and stored at −80°C until needed. Each sample was replicated three times to yield nine samples (three groups × three biological replicates) and the three groups were designated H (HFFs), T (*T. gondii*), and HT (HFFs infected with *T. gondii*).

### Protein Extraction and Quantification

The samples were suspended in a lysis buffer (8 M urea and 1% protease inhibitor) and disrupted three times on ice by sonication. The supernatant was then separated by centrifugation at 4°C and 12,000 *g* for 10 min, and the pellet was removed. Finally, protein concentration was determined using a BCA protein assay kit (catalog no. P0011-1, Beyotime, China), according to manufacturer instructions.

### Trypsin Digestion and TMT Labeling

Dithiothreitol (5 mM, catalog no. D9163, Sigma-Aldrich, USA) was added to the protein solution, and reduction was allowed to proceed at 56°C for 30 min. The protein solution was then incubated with 11 mM iodoacetamide (catalog no. V900335, Sigma-Aldrich, USA) at room temperature in darkness for 15 min. The urea concentration of the solution was then diluted to <2 M using 100 mM tetraethylammonium bromide (catalog no. 17902, Sigma-Aldrich, USA). Finally, trypsin (catalog no. V5111, Promega, USA) was added at a trypsin-to-protein mass ratio of 1:50 for the first round of digestion for overnight and 1:100 for the second round of digestion for 4 h. Peptides were desalted on a Strata X C18 SPE column (Phenomenex, USA) and vacuum dried after trypsin digestion. They were then labeled using a TMT 10-plex isobaric label kit (catalog no. 90111, Thermo, USA), according to manufacturer instructions.

### High-Performance Liquid Chromatography Fractionation and LC-MS/MS

The labeled peptides were fractionated on a 300 Extend C18 column (4.6 mm × 250 mm, 5 μm, Agilent, USA) and separated with a gradient of 8%–32% acetonitrile (pH 9.0) over 60 min into 60 fractions. The peptides samples were then combined into 14 fractions and dried in a vacuum centrifuge. Subsequently, they were dissolved in 0.1% formic acid solution and loaded onto an EASY-nLC 1000 UPLC system (Thermo, USA). Solvent A was 0.1% formic acid in 90% acetonitrile solution. The gradient comprised an increase from 9% to 26% of solvent B (0.1% formic acid in 90% acetonitrile) over 40 min, a rise from 26% to 35% in 14 min, and then from 35% to 80% in 3 min, followed by holding at 80% for the last 4 min; the flow rate was kept constant at 350 nL/min at all times. The peptides were subjected to NSI source, followed by tandem mass spectrometry (MS/MS) on a Q Exactive Plus Orbitrap LC-MS/MS System (Thermo, USA). The electrospray voltage was 2.0 kV. The mass spectrometer scan range was 350–1800 *m/z* and the orbitrap resolution was set to 70,000. The MS/MS scan range and orbitrap resolution were 100 *m/z* and 35,000, respectively. Data-dependent acquisition was used for data collection. Automatic gain control was set to 5E4, and the dynamic exclusion duration was 30 s.

### Database Search

The raw files from the same batch were processed together with MaxQuant against the SwissProt Homo sapiens and UniPort *T. gondii* ATCC_50861 databases, concatenated with the reverse decoy database. The cleavage enzyme was trypsin/P, with no more than two missing cleavages and the minimum peptide length was seven. For precursor ions, the mass tolerance of the first search and main search was set to 20 ppm and 5 ppm, respectively. The mass tolerance of fragment ions was 0.02 Da. Carbamidomethylation of cysteine residues and methionine oxidation were specified as a fixed and variable modification, respectively. The protein quantification method was set with TMT 10-plex and the reporter ion isotopic impurity distribution of TMT 10-plex was set as instructed by to the product manual. The results were filtered based on the peptide false discovery rate (FDR) of < 1%.

### Data Analysis and Bioinformatics

We chose unique peptides for protein quantification; relative quantification of protein expression levels were calculated as the median of unique peptides. There biological replicates were assessed for each group. For each protein, the average ratio calculated using the three biological replicates indicated final protein expression. The protein quantification results were statistically analyzed using two-sample *t*-test and the p-value was corrected by the method of Benjamin and Hochberg FDR analysis. Proteins with a fold change > 1.5 or < 0.67 and adjusted p-value < 0.05 were considered to be differentially abundant. Differentially expressed proteins (DEPs) were selected for subcellular localization, clusters of orthologous groups of protein, gene ontology (GO) classification, and enrichment analyses. WoLF PSORT (v0.2 http://www.genscript.com/psort/wolf_psort.html) was used to predict the subcellular localization of DEPs. For GO analysis, all annotations were derived from the UniProt-GOA database (http://www.ebi.ac.uk/GOA/) and complemented with InterProScan soft (v5.14-53.0 http://www.ebi.ac.uk/InterProScan/). All DEPs were subjected to analysis of eukaryotic orthologous group (KOG) by aligning their sequences with the KOG protein sequence database (http://genome.jgi.doe.gov/help/kogbrowser.jsf). The Kyoto Encyclopedia of Genes and Genomes (KEGG) database was used for pathway enrichment analyses of DEPs. For GO and KEGG enrichment analyses, two-tailed Fisher’s exact test was applied to test DEPs against all identified proteins. A corrected p value of <0.05 indicated statistical significance. MS data have been deposited in the ProteomeXchange Consortium *via* the PRIDE (Perez-Riverol et al., 2019) partner repository, with the dataset identifier PXD021736.

### Parallel Reaction Monitoring (PRM) Validation

To confirm the authenticity and accuracy of results of TMT analysis, we performed PRM assay that included 14 peptides. Protein extraction and digestion were achieved using the aforementioned methods. The peptides were subjected to MS/MS on a Q Exactive Plus Orbitrap LC-MS/MS System (Thermo, USA). The electrospray voltage was 2.1 kV, MS scan range was 350–1050 *m/z*, and orbitrap resolution was 70,000. The MS/MS orbitrap resolution was 17,500. Data-independent acquisition was used for data collection. Automatic gain control of MS and MS/MS was set to 3E6 and 1E5, respectively. The maximum injection time was 50 ms for MS and 180 ms for MS/MS. The isolation window for MS/MS was 1.6 *m/z*. Three biological replicates were assessed.

### Data Analysis of PRM Validation

The resulting MS data were processed using Skyline (v3.6). The peptide settings were as follows: the enzyme was set as trypsin [KR/P] and the max missed cleavage was set as 0. The peptide length was 7–25, and carbamidomethylation of cysteine residues was the fixed modification. The transition settings were as follows: precursor charges were set as 2, 3; ion charges were set as 1; and ion types were set as b, y. The product ions were set as from ion 3 to the last ion, and the ion match tolerance was 0.02 Da.

## Results

### Protein Identification and Expression

**Figure 1:**
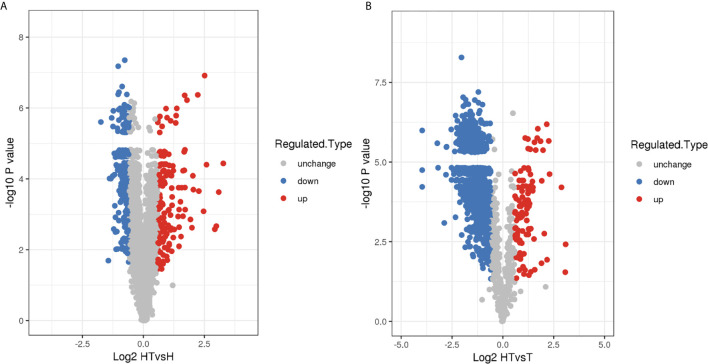
Volcano map of DEPs in the **(A)** HT *vs*. H and **(B)** HT *vs*. T groups.

From all nine samples, 240,537 spectra were generated, from which 26,251 peptides and 25,528 unique peptides were obtained. A total of 4111 and 1708 proteins were identified as human and *T. gondii* proteins, of which 3477 and 1434 proteins were quantified, respectively (**Supplementary Table S1** **)**. The standard criteria were adjusted p-value of <0.05 and > 1.5 or < 0.67 -fold change accordingly, 150 up- and 225 downregulated proteins were identified in the HT group in comparison with the H group (**Figure 1A** and **Supplementary Table S2A**). Further, 107 up- and 992 downregulated proteins were found in the HT group in comparison with the T group (**Figure 1B** and **Supplementary Table S2B**). All DEPs were subsequently subjected to comprehensive bioinformatics analysis.

### Comparative Proteomics Analyses of Host Cells in the HT *vs*. H Group

**Figure 2:**
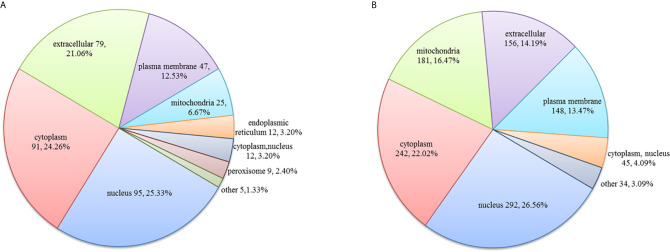
Subcellular location of DEPs in the **(A)** HT *vs*. H and **(B)** HT *vs*. T groups. Numbers represent the number of DEPs.

**Figure 3:**
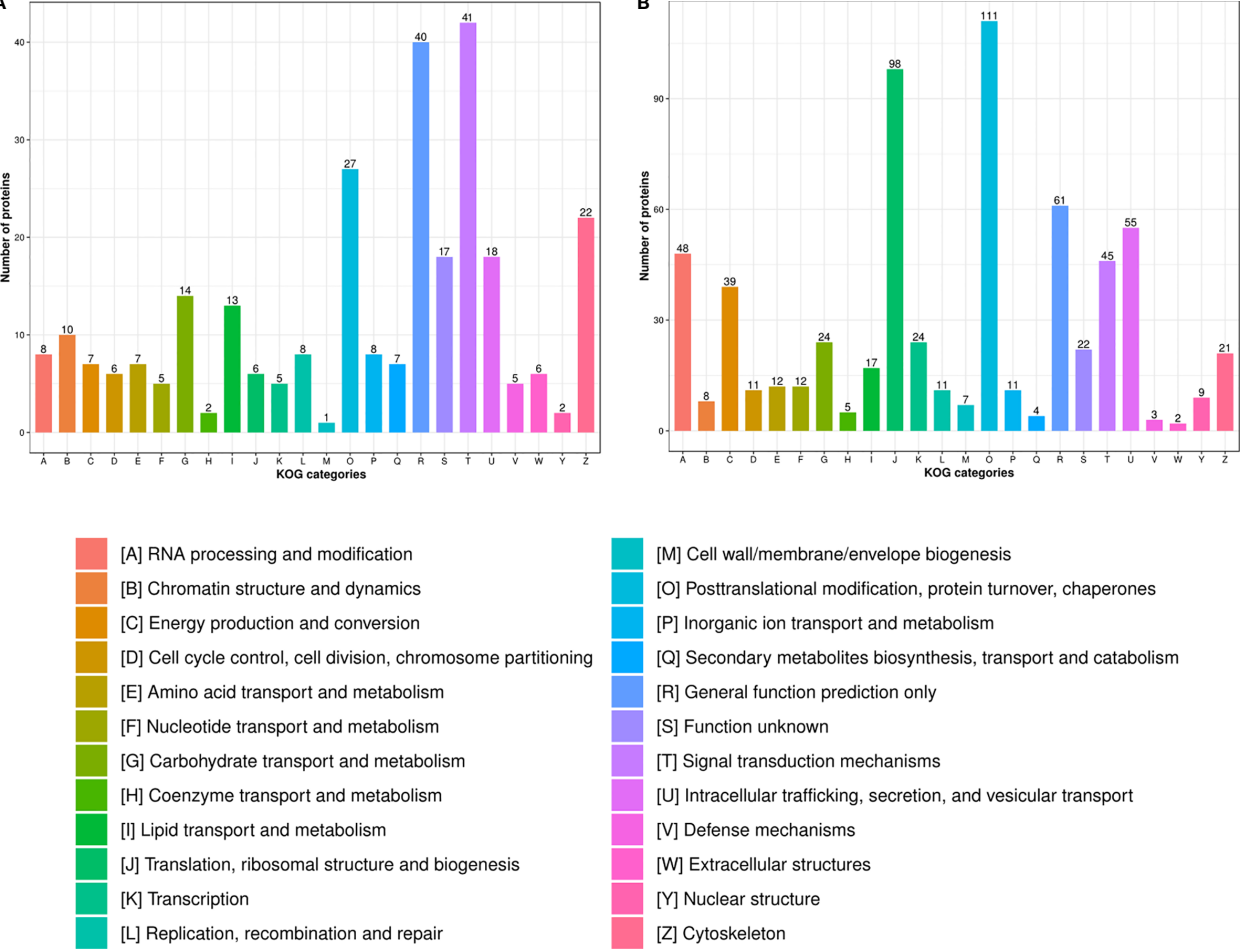
COG analysis of DEPs in the **(A)** HT *vs*. H and **(B)** HT *vs*. T groups. X-axis represents COG/KOG categories. Y-axis represents the numbers of proteins.

In the HT *vs*. H group, the subcellular location of DEPs was as follows: the nucleus (25.33%), cytoplasm (24.26%), and extracellular (21.06%; **Figure 2A**). As evident from **Figure 3A**, DEPs were classified into 24 categories by cluster of orthologous group analysis. The top five categories with the most DEPs were signal transduction mechanisms [T]; general function prediction only [R]; posttranslational modification, protein turnover, chaperones [O]; cytoskeleton [Z]; and intracellular trafficking, secretion, and vesicular transport [U].

**Figure 4:**
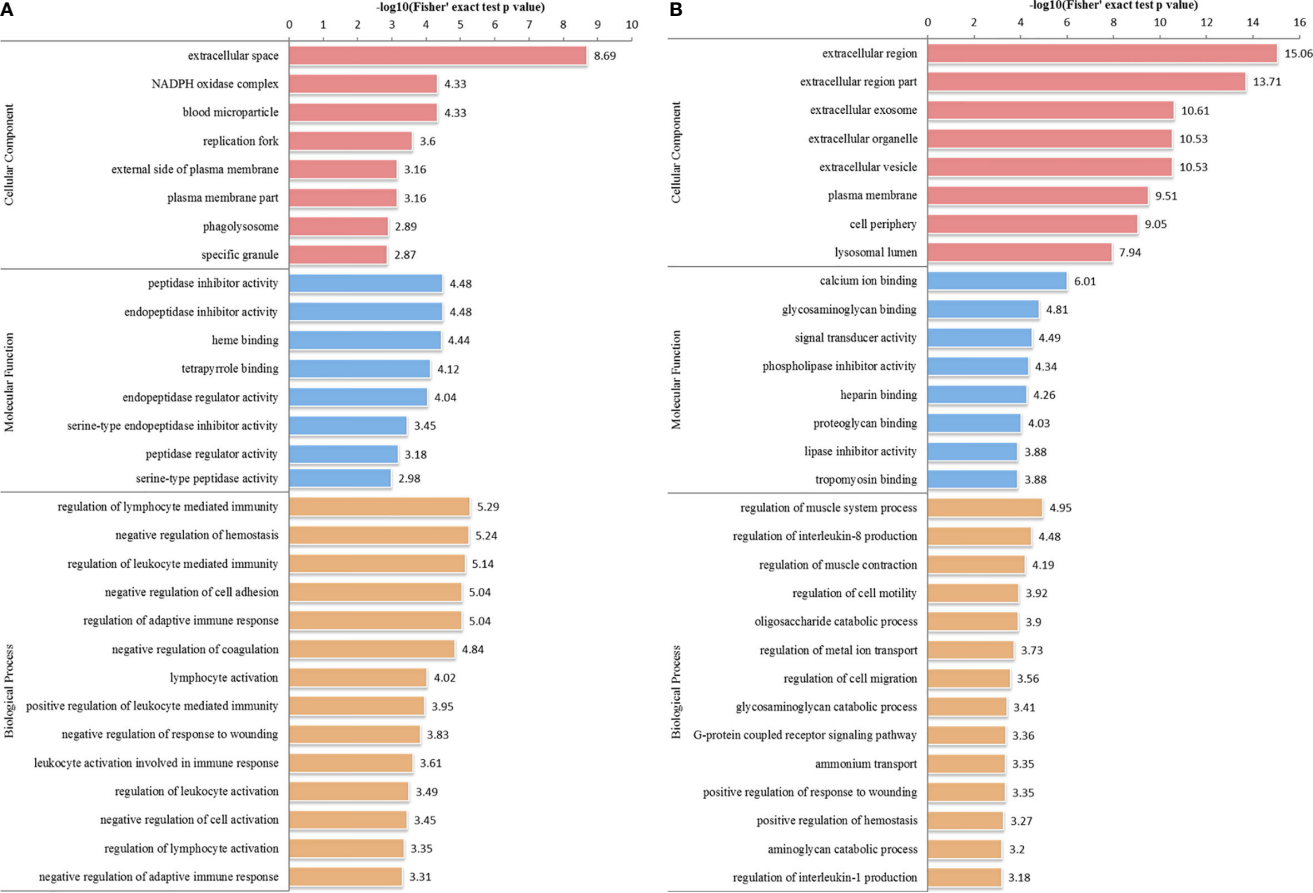
GO distribution of DEPs in the HT *vs*. H group. The enriched GO terms under cellular component, biological process, and molecular function classifications for **(A)** upregulated and **(B)** downregulated proteins.

**Figure 5:**
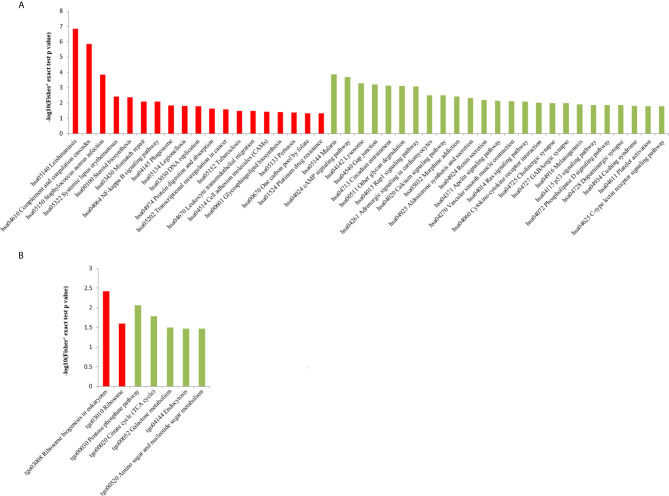
KEGG pathway enrichment analysis of DEPs in the **(A)** HT *vs*. H and **(B)** HT *vs*. T groups. X-axis represents the significantly enriched KEGG pathways. Y-axis represents logarithmic corrected p values [–log10(p)].

We performed GO analysis to study biological changes in infected HFFs in the cellular component, biological process, and molecular function categories. Among the up- and downregulated proteins, 8, 14, and 8 terms in the cellular component, biological process, and molecular function categories were significantly enriched (**Figure 4** and **Supplementary Table S3**), respectively. In the biological process category, the enriched GO terms for immunity/defense-related functions were upregulated, such as regulation of lymphocyte-mediated immunity, regulation of leukocyte-mediated immunity, and regulation of adaptive immune response (**Figure 4A**), whereas those for cell metabolism-related functions were downregulated, such as oligosaccharide catabolic process, glycosaminoglycan catabolic process, and aminoglycan catabolic process (**Figure 4B**). According to KEGG pathway analysis, up- and downregulated proteins were significantly enriched in 19 and 25 pathways, respectively; some immune-related signaling pathways were present among them, such as the NF-κB signaling pathway, cAMP signaling pathway, cell adhesion molecules (CAMs), Rap1 signaling pathway, and calcium signaling pathway (**Figure 5A** and **Supplementary Table S4**).

### Host Proteins Associated With the Immune Response Were Up- and Downregulated Upon Infection With *T. gondii*

*T. gondii* stimulated the host immune system. The expression of proteins associated with the immune response was upregulated, such as apoptotic proteins (Cytochrome c and TNF receptor-associated factor 1), adhesion proteins [intercellular adhesion molecule 1 (ICAM1), programmed cell death 1 ligand 1 (CD274), receptor-type tyrosine-protein phosphatase C (PTPRC), and integrin beta-2 (ITGB2)], and transcriptional regulatory factors (nuclear factor NF-kappa-B p100 subunit and prostaglandin G/H synthase 2). In contrast, the expression of ras-related proteins (rap-1b, m-ras, rab-32, and r-ras2) was downregulated (**Supplementary Table S2A**).

### Signaling Pathways in Host Cells Were Altered During *T. gondii* Infection

**Figure 6:**
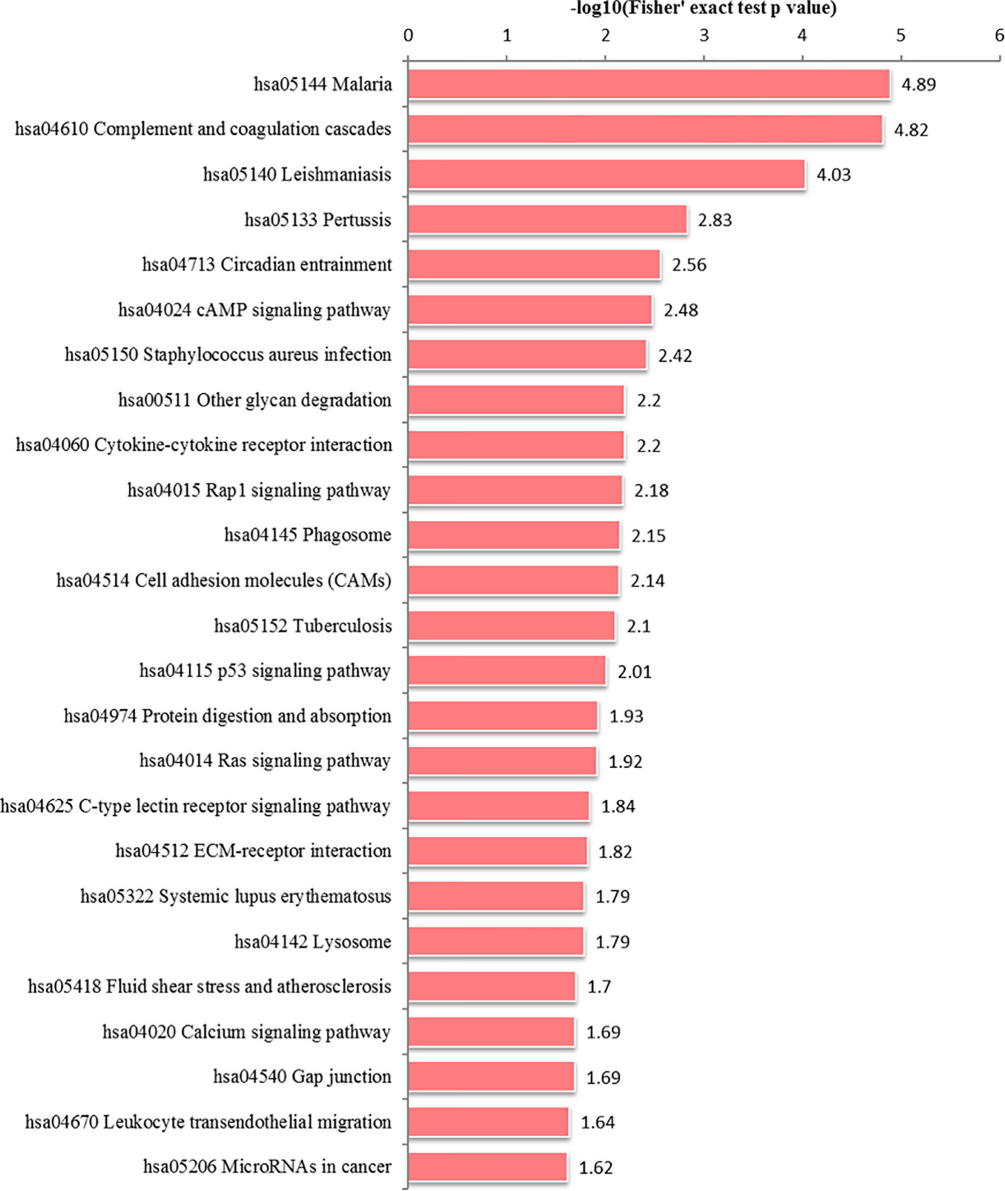
Alteration of signaling pathways in host cells during *T. gondii* infection. X-axis represents significantly enriched KEGG pathways. Y-axis represents logarithmic corrected p values [–log10(p)].

In the HT *vs*. H group, all DEPs could be mapped to 91 pathways, of which 25 were significantly enriched (**Figure 6** and **Supplementary Table S4**). These significantly enriched pathways could be classified into cell metabolism and catabolism pathways (glycan degradation, phagosome and lysosome), immune-related signaling pathways (leukocyte transendothelial migration, calcium signaling pathway, Ras signaling pathway, cell adhesion molecules (CAMs), Rap1 signaling pathway, p53 signaling pathway, cAMP signaling pathway, complement and coagulation cascades) and signaling molecules and interaction pathways (cytokine-cytokine receptor interaction and ECM-receptor interaction).

### Comparative Proteomics Analysis of *T. gondii* in the HT *vs*. T Group

In the HT *vs*. T group, the subcellular location of DEPs was as follows: the nucleus (26.56%), cytoplasm (22.02%), and mitochondria (16.47%; **Figure 2B**). All DEPs were divided into 24 categories by cluster of orthologous group analysis, and the top five categories with the most DEPs were posttranslational modification, protein turnover, chaperones [O]; translation, ribosomal structure, and biogenesis [J]; general function prediction only [R]; intracellular trafficking, secretion, and vesicular transport [U]; and RNA processing and modification [A] (**Figure 3B**).

**Figure 7:**
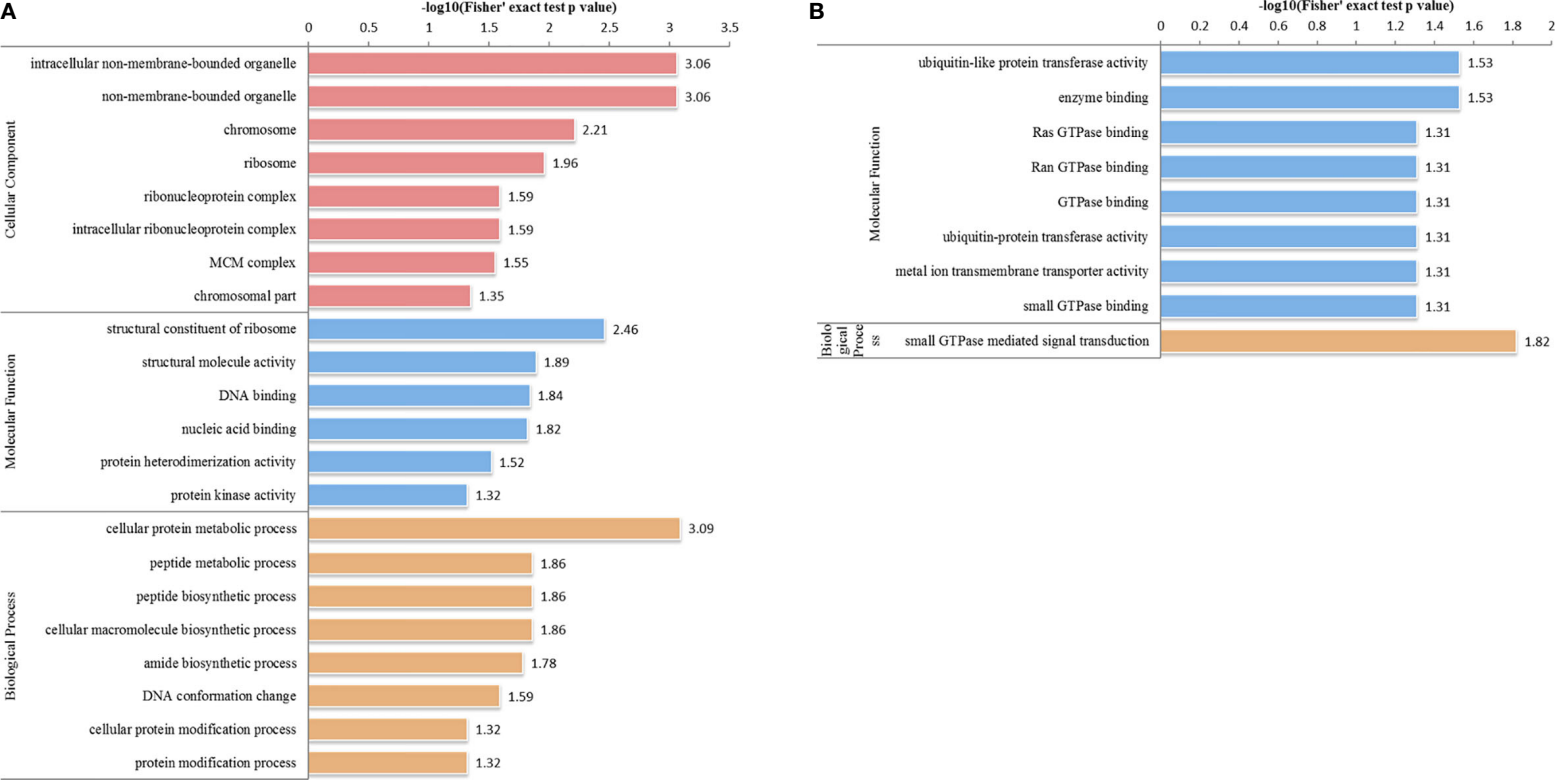
GO analysis of DEPs in the HT *vs*. T group. The enriched GO terms under cellular component, biological process, and molecular function classifications for **(A)** upregulated and **(B)** downregulated proteins.

In the HT *vs*. T group, 1099 DEPs were classified into eight terms of biological process, five terms of cellular component, and five terms of molecular function. Among the upregulated proteins, eight, six, and eight terms for cell component, biological process, and molecular function were significantly enriched, respectively, whereas the downregulated proteins were significantly enriched in one term of biological process and eight terms of molecular function (**Figure 7** and **Supplementary Table S5**). In the biological process category, cell biosynthesis and metabolism were upregulated on infection with *T. gondii*, including cellular protein metabolic, peptide metabolic, and peptide biosynthetic processes (**Figure 7A**). Further, KEGG pathway analysis of the HT *vs*. T group revealed that two significantly enriched pathways were upregulated, including ribosome and ribosome biogenesis in eukaryotes, and six enriched pathways were downregulated, including the pentose phosphate pathway, citrate cycle (TCA cycle), and galactose metabolism (**Figure 5B** and **Supplementary Table S6**).

### *T. gondii* Infection Altered the Expression of Numerous Secretory Proteins

*T. gondii* invasion relies on the secretion of numerous secretory proteins, which mainly originate from three secretory organelles: micronemes, rhoptries, and dense granules. In the HT *vs*. T group, the expression of few secretory proteins was upregulated, such as MICs (MIC6, MIC10), ROPs (ROP5, ROP17), and GRAs (GRA4, GRA5, and GRA12). In contrast, the expression of dozens of known secretory proteins was significantly downregulated in *T. gondii*-infected HFFs (**Table 1**). As shown in **Table 1**, 10 SAG1-related sequence proteins (SRSs) exhibited a 0.67-fold or lower decrease in their expression levels in the HT *vs*. T group, whereas four SRSs exhibited a 1.5-fold or greater increase in their expression levels, including SRS29A (2.938 fold), SRS36D (1.919 fold), SRS57 (1.769 fold), and SRS20A (1.524 fold).

**Table 1:** Details pertaining to differentially expressed secretory proteins.

Protein accession	Protein description	Gene name	HT/T ratio	Regulated type	HT/T p value
**B9Q4G2**	SAG-related sequence SRS29A	BN1205_022090	2.938	Up	2.39E-06
**B9PRE4**	Microneme protein 10	BN1205_001390	2.008	Up	3.79E-05
**V5BF07**	SAG-related sequence SRS36D	TGVEG_292280	1.919	Up	3.80E-05
**B6KB88**	Rhoptry kinase family protein ROP17	BN1205_075890	1.781	Up	3.40E-03
**A0A0F7V6G5**	SAG-related sequence SRS57	BN1205_069910	1.769	Up	5.64E-04
**B9QLZ0**	Microneme protein MIC6	BN1205_071220	1.752	Up	8.44E-03
**V4Z6U4**	Rhoptry protein ROP5	TGVEG_308090	1.59	Up	4.40E-02
**B9QGZ4**	SAG-related sequence SRS20A	BN1205_033260	1.524	Up	3.61E-04
**A0A125YSX9**	Rhoptry neck protein RON5	TGVEG_311470	0.658	Down	2.22E-06
**B9PUW8**	Microneme protein MIC11	BN1205_044690	0.65	Down	5.41E-04
**V5B190**	Rhoptry kinase family protein ROP26	TGVEG_211260	0.636	Down	2.31E-05
**A0A125YX19**	Microneme protein MIC7	TGVEG_261780	0.629	Down	4.30E-05
**A0A125YRV5**	Rhoptry neck protein RON8	TGVEG_306060	0.585	Down	1.04E-04
**V5BDH5**	SAG-related sequence SRS38B	BN1205_085760	0.585	Down	1.93E-05
**V4Z883**	Microneme protein, putative	BN1205_055460	0.568	Down	1.24E-03
**B9QHX6**	Rhoptry protein ROP15	BN1205_009170	0.538	Down	5.86E-05
**A0A0F7UVV5**	Rhoptry kinase family protein ROP16	BN1205_071990	0.534	Down	5.79E-05
**V4ZLN9**	SAG-related sequence SRS30A	TGVEG_273130	0.528	Down	1.82E-04
**A0A0F7UZH2**	Microneme protein 2	BN1205_047640	0.522	Down	6.22E-07
**B6KK34**	SAG-related sequence SRS29C	BN1205_022060	0.516	Down	5.71E-05
**A0A125YGR1**	Rhoptry kinase family protein ROP40	TGVEG_291960	0.5	Down	2.28E-05
**B6KB96**	Rhoptry protein 6	BN1205_075810	0.494	Down	6.50E-05
**B9QR15**	Rhoptry kinase family protein ROP35	BN1205_041270	0.489	Down	3.14E-03
**B9Q846**	SAG-related sequence SRS67	BN1205_067870	0.48	Down	5.86E-07
**V4YW47**	Rhoptry neck protein RON3	BN1205_064400	0.477	Down	5.25E-04
**B6KL08**	Microneme protein MIC1	BN1205_084920	0.476	Down	5.59E-04
**B9QFH2**	Rhoptry protein ROP12	BN1205_045220	0.466	Down	2.01E-05
**B9PXJ3**	Microneme protein MIC4	BN1205_040160	0.453	Down	3.63E-05
**A0A0F7V1Z5**	SAG-related sequence SRS34A	BN1205_018330	0.421	Down	1.84E-05
**V4Z9F7**	Rhoptry kinase family protein ROP39	BN1205_072800	0.414	Down	3.19E-04
**A0A125YXY8**	Putative rhoptry protein	TGVEG_315210	0.403	Down	5.65E-04
**V4Z8E3**	Microneme protein MIC17A	TGVEG_200250	0.389	Down	1.66E-05
**A0A125YHB4**	Microneme protein MIC5	TGVEG_277080	0.381	Down	3.89E-05
**V4YND8**	Microneme protein MIC3	TGVEG_319560	0.379	Down	4.41E-06
**B9Q6P5**	SAG-related sequence SRS43	BN1205_062310	0.369	Down	1.54E-05
**A0A0F7V8G6**	Rhoptry protein ROP13	BN1205_096370	0.354	Down	1.89E-05
**B6KDK1**	SAG-related sequence SRS30C	BN1205_020430	0.326	Down	3.85E-05
**B9QEX1**	SAG-related sequence SRS44	BN1205_081610	0.314	Down	1.54E-05
**B9PSP5**	SAG-related sequence SRS25	BN1205_035070	0.302	Down	4.98E-07
**V4ZDA0**	Rhoptry protein ROP18	TGVEG_205250	0.301	Down	1.78E-04
**B6K9I7**	SAG-related sequence SRS52A	BN1205_100430	0.285	Down	1.01E-04
**A0A0F7VCB9**	Microneme protein 8	BN1205_006390	0.231	Down	1.64E-05
**V5BFN0**	Microneme protein MIC15	BN1205_004820	0.174	Down	2.93E-06

### Validation of DEPs

We performed PRM assay for an independent comparison and validation of the results of TMT analysis. In the HT *vs*. H group, six proteins—desmoplakin protein (DSP), plexin-B2 (PLXNB2), annexin A1 (ANXA1), early endosome antigen 1 (EEA1), DNA topoisomerase 1 (TOP1), and probable ATP-dependent RNA helicase (DDX5)—were selected for analysis. As evident from **Table 2**, the results of PRM assay were consistent with those of TMT analysis. In case of the HT *vs*. T group, we selected MIC1, MIC2, ROP5, GRA12, and two heat shock proteins (HSP28 and HSP90) for PRM assay. The expression level of MIC1, MIC2, and HSP90 was downregulated and that of ROP5, GRA12, and HSP28 was upregulated, validating the consistency of results obtained using the two methods (**Table 2** and **Supplementary Table S7**).

**Table 2:** Comparison of the quantification results of TMT analysis and PRM assay of candidate DEPs in the HT *vs*. H and HT *vs*. T groups.

Protein accession	Protein gene	Protein description	Peptide sequence	PRM ratio	p value	TMT ratio	p value
O15031	PLXNB2	Plexin-B2	LVECGSLFK	0.50	2.48E-04	0.61	2.49E-06
P04083	ANXA1	Annexin A1	GVDEATIIDILTK	0.54	3.36E-05	0.62	1.89E-05
Q15075	EEA1	Early endosome antigen 1	EQALQDLQQQR	0.58	1.54E-04	0.67	1.72E-05
P11387	TOP1	DNA topoisomerase 1	ELTAPDENIPAK	1.49	4.80E-03	1.60	1.73E-06
P17844	DDX5	Probable ATP-dependent RNA helicase DDX5	NFYQEHPDLAR DWVLNEFK	1.80	2.66E-06	1.51	2.18E-06
P15924	DSP	Desmoplakin	LPVDIAYK	1.19	5.71E-02	1.59	1.78E-05
A0A0F7V6Y1	HSP90	Heat shock protein 90	EDQTEYLEDR	0.57	3.24E-04	0.64	9.44E-04
A0A0F7UZH2	MIC2	Microneme protein 2	NPWNEDQQHGGLSCEQQHPGGR	0.65	4.60E-03	0.52	2.28E-05
B6KL08	MIC1	Microneme protein MIC1	HYTEEEGIR	0.57	7.91E-04	0.48	1.84E-05
V4Z6U4	ROP5	Rhoptry protein ROP5	EEELIGYCR	1.88	6.89E-04	1.59	4.40E-02
B9PSE4	HSP28	Heat shock protein 28	DHGEEDFVR	48.02	1.01E-07	2.96	2.34E-02
B9QDT9	GRA12	Dense granule protein GRA12	AATVAAGNELFK ASETGSGLAASFLNTVEVR	11.02	1.53E-05	1.59	2.74E-03

## Discussion

With technological advancements, mass spectrometry-based proteomic approaches have rapidly developed; compared to two-dimensional electrophoresis and difference gel electrophoresis, they are high throughput methods for protein identification and quantification. Nelson et al. (2008) identified 157 DEPs in HFFs before and after infection with *T. gondii* using two-dimensional electrophoresis and mass spectrometry, whereas we herein identified 1400 DEPs (adjusted p-value < 0.05 and >1.2 or <0.83 fold change, **Supplementary Table S1A**) in response to infection with *T. gondii* using TMT analysis. The host demonstrated global reprogramming of cell metabolism upon *T. gondii* invasion, as evident from the differential expression levels of proteins involved in key metabolic pathways (glycolysis, lipid and sterol metabolism, mitosis, apoptosis, and structural protein expression). In the present study, we focused on host cell signaling pathways related to cell survival, innate recognition of parasites, and immune response.

In the process of infecting hosts, parasites secrete various proteins to regulate the host signal transduction mechanism and modulate the immune response of host cells (Shapira et al., 2005; Muniz-Feliciano et al., 2013). In this study, immune-related signaling pathways were significantly enriched, such as the NF-κB, cAMP, Rap1, Ras, cytokine–cytokine receptor interaction, p53, platelet activation, and MAPK signaling pathways (**Supplementary Table S4**), suggesting that the innate and adaptive immunity of the host was majorly modulated in response to *T. gondii* infection.

As obligate intracellular parasites, *T. gondii* must traverse the host cell plasma membrane to initiate an infection and to ensure survival and replication. *T. gondii* invasion and infection establishment relies on secretory proteins, including MICs, ROPs, RONs, GRAs, and SRSs (Manger et al., 1998; Carey et al., 2000; Lekutis et al., 2001; Tomley and Soldati, 2001; Mercier et al., 2002; Bradley et al., 2005). The majority of MICs comprise multiple copies of a conserved adhesive domain, allowing them to bind to host cell surface receptors or carbohydrates during pathogen invasion. Carruthers and Tomley (2008) indicated that MIC secretion is rapidly upregulated when parasites make contact with host cells. We herein observed that the expression levels of MIC6 and MIC10 were upregulated, but those of most other MICs (MIC1–5, MIC7, MIC8, MIC11, MIC15, MIC17A, and M2AP) were downregulated; this could be because we analyzed the proteome at a single timepoint. In addition, the adhesion-related domains of MICs not only adhere to host cells but also promote complex formation between different MICs, making them more stable on the *T. gondii* surface and increasing its invasive capability. Multiple MICs have been previously reported, including MIC1–MIC4–MIC6, MIC2–M2AP, and MIC3–MIC8; all complex components were downregulated, except MIC6, in the current study. GRA6 can evidently activate the host transcription factor NFAT4, which promotes the synthesis of the chemokines Cxcl2 and Ccl2, and chemokines are known to control the spread of parasites (Ma et al., 2014; Hakimi et al., 2017). In this study, GRA6 expression level was downregulated after host cell invasion, consequently facilitating *T. gondii* survival. It has been reported that many SRSs are stage-specific; they play a key role in mediating the attachment to host cells and activate host immunity to regulate *T. gondii* virulence (Jung et al., 2004). For example, SRS57 serves as an adhesion protein by binding to the host cell surface, SRS29B is a virulence factor, and SRS34A and SRS16B play a crucial role in host cell immune modulation (Dzierszinski et al., 2000; Van et al., 2007; Tomita et al., 2018). In the HT group, the expression level of many SRS proteins, including, SRS29A, SRS36D, SRS57, and SRS20A, was upregulated and that of others (SRS38B, SRS30A, SRS29C, SRS67, SRS34A, SRS43, SRS30C, SRS44, SRS25, and SRS52A) was downregulated in comparison with the expression levels in the T group. So far, few studies exist on SRSs, and the understanding of their various biological functions needs to be enhanced in future.

Parasite invasion is an active process that relies on the cytoskeleton for motility and also on the host cytoskeleton (Dobrowolski and Sibley, 1996). Host microtubules have been observed to play a role in interactions with invading *T. gondii* tachyzoites, and the host cytoskeleton apparently contributes to the anchoring of the moving junction (Sweeney et al., 2010; Takemae et al., 2013). Microtubules are composed of heterodimers of alpha- and beta-tubulin. Takemae et al. (2013) reported that TUBB2C and TUBB5 interact with TgRON4. In our study, TUBB2C and TUBB5 were not identified as DEPs in the HT *vs*. H group, but other alpha- and beta-tubulin proteins were detected, including TUBA1C (0.625 fold), TUBA4A (0.595 fold), TUBB2A (0.801 fold), TUBB3 (0.688 fold), TUBB4B (0.646 fold), and TUBB6 (0.753 fold) (**Supplementary Table S1A**), indicating that infection with *T. gondii* causes the remodeling of the host cytoskeleton.

CAMs are distributed throughout regions of the plasma membrane and come in contact with other cells and extracellular matrix (Figliuolo da Paz et al., 2019). Herein the pathways of CAMs were significantly enriched and included neuronal growth regulator 1, ICAM1, syndecan-2, CD274, neural cell adhesion molecule L1, PTPRC, junctional adhesion molecule C, and ITGB2. ICAM1 is expressed on various cell types and participates in diverse cellular processes, including host–pathogen interactions (Bhalla et al., 2015). ICAM1 has been reported to interact with PfEMP1 in *Plasmodium falciparum* infection (Lennartz et al., 2019). Further, ICAM1 expression level has been noted to be upregulated upon host cell invasion by *T. gondii in vitro* and also during human toxoplasma uveitis *in vivo*, with ICAM1 interacting with TgMIC2 (Klok et al., 1999; Nagineni et al., 2000; Barragan et al., 2005). In this study, compared with uninfected HFFs, ICAM1 expression in infected cells was upregulated (1.96 fold). Blader et al. (2001) demonstrated that the ICAM1 gene was upregulated by at least 2-fold in HFFs after *T. gondii* invasion, and Nagineni et al. (2000) reported that ICAM1 secretion in human retinal pigment epithelial cells infected with *T. gondii* was augmented 5-fold over the uninfected control, and no significant changes in ICAM1 levels were found in human retinal pigment epithelial cells incubated with heat-killed *T. gondii*. These findings suggest that ICAM1 expression is derived from the interaction between host cells and the parasite.

Based on their roles in host–pathogen interaction, Spear et al. (2006) clustered host genes into three distinct classes: “pro-host”, host genes required for host defense; “pro-parasite”, host genes required for parasite growth; and “bystander”, host genes incidentally regulated as a consequence of modulating the first two classes. In present study, we performed TMT-based proteomic analysis of *T. gondii* as well as host cells at 24 h of infection. The infection induced significant changes in the proteome of *T. gondii* and also led to the significant enrichment of immune-related signaling pathways in HFFs. Various DEPs, including cytoskeleton and immune-related proteins, were induced in response to host cell invasion, indicating that infection with *T. gondii* leads to considerable remodeling of the host cytoskeleton and also the crucial role played by the host immune system upon encountering *T. gondii*. Future studies are warranted to validate these proteins and to assess their clinical value.

## Data Availability Statement

The datasets presented in this study can be found in online repositories. The names of the repository/repositories and accession number(s) can be found in the article/**Supplementary Material**.

## Author Contributions

HS and JC designed this study and made the final revision. HS, JL and LW performed the experiments. KY, CX, GL and TX helped to create the tables and figures. BH and QW contributed to the reagents and materials. MG supervised the process. HS wrote the draft manuscript and JC revised the manuscript. All authors contributed to the article and approved the submitted version.

## Funding

This work was supported by grants from the Key Research and Development Program of Shandong Province (Grant No. 2019GSF107054 to HS), the National Natural Science Foundation of China (Grant No. 81501770 to HS), NHC Key Laboratory of Parasite and Vector Biology (Grant No. WSBKFKT201805 to HS), and the Fifth Round of Three-Year Public Health Action Plan of Shanghai (No. GWV-10.1-XK13 to JC). The funders had no role in the study design, data collection and analysis, decision to publish, or preparation of the manuscript.

## Conflict of Interest

The authors declare that the research was conducted in the absence of any commercial or financial relationships that could be construed as a potential conflict of interest.

## References

[B1] Barragan A. Brossier F. Sibley L. D. (2005). Transepithelial Migration of *Toxoplasma Gondii* Involves an Interaction of Intercellular Adhesion Molecule 1 (ICAM-1) With the Parasite Adhesin MIC2. Cell Microbiol. 7, 561–568. 10.1111/j.1462-5822.2005.00486.x 15760456

[B2] Bhalla K. Chugh M. Mehrotra S. Rathore S. Tousif S. Prakash D. V.. (2015). Host ICAMs Play a Role in Cell Invasion by *Mycobacterium Tuberculosis* and *Plasmodium Falciparum* . Nat. Commun. 6, 6049. 10.1038/ncomms7049 25586702

[B3] Blader I. J. Manger I. D. Boothroyd J. C. (2001). Microarray Analysis Reveals Previously Unknown Changes in Toxoplasma Gondii-Infected Human Cells. J. Biol. Chem. 276 (26), 24223–24231. 10.1074/jbc.M100951200 11294868

[B4] Bradley P. J. Ward C. Cheng S. J. Alexander D. L. Coller S. Coombs G. H.. (2005). Proteomic Analysis of Rhoptry Organelles Reveals Many Novel Constituents for Host-Parasite Interactions in *Toxoplasma Gondii* . J. Biol. Chem. 280, 34245–34258. 10.1074/jbc.M504158200 16002398

[B5] Carey K. L. Donahue C. G. Ward G. E. (2000). Identification and Molecular Characterization of GRA8, a Novel, Proline-Rich, Dense Granule Protein of *Toxoplasma Gondii* . Mol. Biochem. Parasitol. 105, 25–37. 10.1016/s0166-6851(99)00160-7 10613696

[B6] Carruthers V. B. Tomley F. M. (2008). Microneme Proteins in Apicomplexans. Subcell. Biochem. 47, 33–45. 10.1007/978-0-387-78267-62 18512339 PMC2847500

[B7] Cohen A. M. Rumpel K. Coombs G. H. Wastling J. M. (2002). Characterisation of Global Protein Expression by Two-Dimensional Electrophoresis and Mass Spectrometry: Proteomics of *Toxoplasma Gondii* . Int. J. Parasitol. 32, 39–51. 10.1016/s0020-7519(01)00308-3 11796121

[B8] Dobrowolski J. M. Sibley L. D. (1996). *Toxoplasma* Invasion of Mammalian Cells is Powered by the Actin Cytoskeleton of the Parasite. Cell 84, 933–939. 10.1016/s0092-8674(00)81071-5 8601316

[B9] Dzierszinski F. Mortuaire M. Cesbron-Delauw M. F. Tomavo S. (2000). Targeted Disruption of the Glycosylphosphatidylinositol-Anchored Surface Antigen SAG3 Gene in *Toxoplasma Gondii* Decreases Host Cell Adhesion and Drastically Reduces Virulence in Mice. Mol. Microbiol. 37, 574–582. 10.1046/j.1365-2958.2000.02014.x 10931351

[B10] Figliuolo da Paz V. R. Figueiredo-Vanzan D. Dos, Santos, Pyrrho A. (2019). Interaction and Involvement of Cellular Adhesion Molecules in the Pathogenesis of Schistosomiasis Mansoni. Immunol. Lett. 206, 11–18. 10.1016/j.imlet.2018.11.011 30503821

[B11] Flegr J. (2013). How and Why *Toxoplasma* Makes Us Crazy. Trends Parasitol. 29, 156–163. 10.1016/j.pt.2013.01.007 23433494

[B12] Frénal K. Polonais V. Marq J. B. Stratmann R. Limenitakis J. Soldati-Favre D. (2010). Functional Dissection of The Apicomplexan Glideosome Molecular Architecture. Cell Host Microbe 8, 343–357. 10.1016/j.chom.2010.09.002 20951968

[B13] Hakimi M. A. Olias P. Sibley L. D. (2017). *Toxoplasma* Effectors Targeting Host Signaling and Transcription. Clin. Microbiol. Rev. 30, 615–645. 10.1128/CMR.00005-17 28404792 PMC5475222

[B14] Janouškovec J. Tikhonenkov D. V. Burki F. Howe A. T. Kolísko M. Mylnikov A. P.. (2015). Factors Mediating Plastid Dependency and the Origins of Parasitism in Apicomplexans and Their Close Relatives. P. Natl. Acad. Sci. U. S. A. 112, 10200–102007. 10.1073/pnas.1423790112 PMC4547307 25717057

[B15] Jiao F. Zhang D. Jiang M. Mi J. Liu X. Zhang H.. (2017). Label-Free Proteomic Analysis of Placental Proteins During *Toxoplasma Gondii* Infection. J. Proteomics. 150, 31–39. 10.1016/j.jprot.2016.08.013 27569050

[B16] Jung C. Lee C. Y. Grigg M. E. (2004). The SRS Superfamily of *Toxoplasma* Surface Proteins. Int. J. Parasitol. 34, 285–296. 10.1016/j.ijpara.2003.12.004 15003490

[B17] Kim K. Weiss L. M. (2008). Toxoplasma: The Next 100 Years. Microbes Infect. 10, 978–984. 10.1016/j.micinf.2008.07.015 18672085 PMC2596634

[B18] Klok A. M. Luyendijk L. Zaal M. J. Rothova A. Kijlstra A. (1999). Soluble ICAM-1 Serum Levels in Patients With Intermediate Uveitis. Br. J. Ophthalmol. 83, 847–851. 10.1136/bjo.83.7.847 10381673 PMC1723107

[B19] Lekutis C. Ferguson D. J. Grigg M. E. Camps M. Boothroyd J. C. (2001). Surface Antigens of *Toxoplasma Gondii*: Variations on a Theme. Int. J. Parasitol. 31, 1285–1292. 10.1016/s0020-7519(01)00261-2 11566296

[B20] Lennartz F. Smith C. Craig A. G. Higgins M. K. (2019). Structural Insights Into Diverse Modes of ICAM-1 Binding by *Plasmodium Falciparum*-Infected Erythrocytes. P. Natl. Acad. Sci. U. S. A. 116, 20124–20134. 10.1073/pnas.1911900116 PMC6778195 31527263

[B21] Manger I. D. Hehl A. B. Boothroyd J. C. (1998). The Surface of *Toxoplasma* Tachyzoites is Dominated by a Family of Glycosylphosphatidylinositol-Anchored Antigens Related to SAG1. Infect. Immun. 66, 2237–2244. 10.1128/IAI.66.5.2237-2244.1998 9573113 PMC108187

[B22] Ma J. S. Sasai M. Ohshima J. Lee Y. Bando H. Takeda K.. (2014). Selective and Strain-Specific NFAT4 Activation by the *Toxoplasma Gondii* Polymorphic Dense Granule Protein GRA6. J. Exp. Med. 211, 2013–2032. 10.1084/jem.20131272 25225460 PMC4172224

[B23] Mercier C. Dubremetz J. F. Rauscher B. Lecordier L. Sibley L. D. Cesbron-Delauw M. F. (2002). Biogenesis of Nanotubular Network in *Toxoplasma* Parasitophorous Vacuole Induced by Parasite Proteins. Mol. Biol. Cell. 13, 2397–2409. 10.1091/mbc.e02-01-0021 12134078 PMC117322

[B24] Montoya J. G. Liesenfeld O. (2004). Toxoplasmosis. Lancet 363, 1965–1976. 10.1016/S0140-6736(04)16412-X 15194258

[B25] Muniz-Feliciano L. Van Grol J. Portillo J. A. Liew L. Liu B. Carlin C. R.. (2013). *Toxoplasma Gondii*-Induced Activation of EGFR Prevents Autophagy Protein-Mediated Killing of the Parasite. PloS Pathog. 9, e1003809. 10.1371/journal.ppat.1003809 24367261 PMC3868508

[B26] Nagineni C. N. Detrick B. Hooks J. J. (2000). *Toxoplasma Gondii* Infection Induces Gene Expression and Secretion of Interleukin 1 (IL-1), IL-6, Granulocyte-Macrophage Colony-Stimulating Factor, and Intercellular Adhesion Molecule 1 by Human Retinal Pigment Epithelial Cells. Infect. Immun. 68, 407–410. 10.1128/iai.68.1.407-410.2000 10603418 PMC97151

[B27] Nelson M. Jones A. R. Carmen J. C. Sinai A. P. Burchmore R. Wastling J. M. (2008). Modulation of the Host Cell Proteome by the Intracellular Apicomplex an Parasite *Toxoplasma Gondii* . Infect. Immun. 76, 828–844. 10.1128/IAI.01115-07 17967855 PMC2223483

[B28] Perez-Riverol Y. Csordas A. Bai J. Bernal-Llinares M. Hewapathirana S. Kundu D. J.. (2019). The PRIDE Database and Related Tools and Resources in 2019: Improving Support for Quantification Data. Nucleic. Acids Res. 47 (D1), D442–D450. 10.1093/nar/gky1106 30395289 PMC6323896

[B29] Rabenau K. E. Sohrabi A. Tripathy A. Reitter C. Ajioka J. W. Tomley F. M.. (2001). TgM2AP Participates in *Toxoplasma Gondii* Invasion of Host Cells and is Tightly Associated With the Adhesive Protein Tgmic2. Mol. Microbiol. 41, 537–547. 10.1046/j.1365-2958.2001.02513.x 11532123

[B30] Robert-Gangneux F. Murat J. B. Fricker-Hidalgo H. Brenier-Pinchart M. P. Gangneux J. P. Pelloux H. (2011). The Placenta: A Main Role in Congenital Toxoplasmosis? Trends Parasitol. 27, 530–536. 10.1016/j.pt.2011.09.005 22079164

[B31] Shapira S. Harb O. S. Margarit J. Matrajt M. Han J. Hoffmann A.. (2005). Initiation and Termination of NF-kappaB Signaling by the Intracellular Protozoan Parasite *Toxoplasma Gondii* . J. Cell. Sci. 118, 3501–3508. 10.1242/jcs.02428 16079291

[B32] Soldati D. Dubremetz J. F. Lebrun M. (2001). Microneme Proteins: Structural and Functional Requirements to Promote Adhesion and Invasion by the Apicomplexan Parasite *Toxoplasma Gondii* . Int. J. Parasitol. 31, 1293–1302. 10.1016/s0020-7519(01)00257-0 11566297

[B33] Spear W. Chan D. Coppens I. Johnson R. S. Giaccia A. Blader I. J. (2006). The Host Cell Transcription Factor Hypoxia-Inducible Factor 1 is Required for *Toxoplasma Gondii* Growth and Survival At Physiological Oxygen Levels. Cell Microbiol. 8, 339–352. 10.1111/j.1462-5822.2005.00628.x 16441443

[B34] Sweeney K. R. Morrissette N. S. LaChapelle S. Blader I. J. (2010). Host Cell Invasion by *Toxoplasma Gondii* is Temporally Regulated by the Host Microtubule Cytoskeleton. Eukaryot. Cell 9, 1680–1689. 10.1128/EC.00079-10 20435700 PMC2976295

[B35] Swierzy I. J. Händel U. Kaever A. Jarek M. Scharfe M. Schlüter D.. (2017). Divergent Co-Transcriptomes of Different Host Cells Infected With *Toxoplasma Gondii* Reveal Cell Type-Specific Host-Parasite Interactions. Sci. Rep. 7, 7229. 10.1038/s41598-017-07838-w 28775382 PMC5543063

[B36] Takemae H. Sugi T. Kobayashi K. Gong H. Ishiwa A. Recuenco F. C.. (2013). Characterization of the Interaction Between *Toxoplasma Gondii* Rhoptry Neckprotein 4 and Host Cellular β-Tubulin. Sci. Rep. 3, 3199. 10.1038/srep03199 24217438 PMC3824165

[B37] Tomita T. Ma Y. Weiss L. (2018). Characterization of a SRS13: A New Cyst Wall Mucin-Like Domain Containing Protein. Parasitol. Res. 117, 2457–2466. 10.1007/s00436-018-5934-3 29911257 PMC6269225

[B38] Tomley F. M. Soldati D. S. (2001). Mix and Match Modules: Structure and Function of Microneme Proteins in Apicomplexan Parasites. Trends Parasitol. 17, 81–88. 10.1016/s1471-4922(00)01761-x 11228014

[B39] Torrey E. F. Yolken R. H. (2013). *Toxoplasma* Oocysts as a Public Health Problem. Trends Parasitol. 29, 380–384. 10.1016/j.pt.2013.06.001 23849140

[B40] Van T. T. Kim S.-K. Camps M. Boothroyd J. C. Knoll L. J. (2007). The BSR4 Protein is Up-Regulated in *Toxoplasma Gondii* Bradyzoites, However the Dominant Surface Antigen Recognised by the P36 Monoclonal Antibody is SRS9. Int. J. Parasitol. 37, 877–885. 10.1016/j.ijpara.2007.02.001 17368655

[B41] Zhang H. Guo F. Zhou H. Zhu G. (2012). Transcriptome Analysis Reveals Unique Metabolic Features in the *Cryptosporidium Parvum* Oocysts Associated With Environmental Survival and Stresses. BMC Genomics 13:647. 10.1186/1471-2164-13-647 23171372 PMC3542205

